# Prognostic Impact of Qualitative and Quantitative Mitral Valve Calcification in Transapical Transcatheter Mitral Valve Replacement: A Sub-Analysis of the TENDER Registry

**DOI:** 10.3390/jcm15072660

**Published:** 2026-03-31

**Authors:** Tillmann Kerbel, Liliane Zillner, Mirjam G. Wild, Michaela M. Hell, Elmar W. Kuhn, Tanja Rudolph, Lenard Conradi, Andreas Zierer, Francesco Maisano, Marco Russo, Fabrizio Rosati, Andrea Colli, Miguel Piñón, David Reineke, Gaby Aphram, Christophe Dubois, Jörg Hausleiter, Ralph Stephan von Bardeleben, Martin Andreas

**Affiliations:** 1 Department of Cardiac and Thoracic Aortic Surgery, Medical University of Vienna, 1090 Vienna, Austria; 2 3rd Medical Department, Cardiology with Intensive Care Medicine, Clinic Ottakring, 1160 Vienna, Austria; 3 Department of Cardiology and Angiology, University Heart Center Freiburg/Bad Krozingen, 79189 Bad Krozingen, Germany; 4 Medizinische Klinik I, LMU University Hospital, 80336 Munich, Germany; 5 Department of Cardiology, University Medical Center Mainz, 55131 Mainz, Germany; 6 Department of Cardiothoracic Surgery, University Hospital Cologne, 50937 Cologne, Germany; 7 Department of Cardiology, Heart-und Diabetes Center Northrhine-Westfalia, Ruhr-University Bochum and University of Bielefeld, 32545 Bad Oeynhausen, Germany; 8 Department for Cardiac-, Vascular- and Thoracic Surgery, Kepler University Hospital, Johannes Kepler University Linz, 4040 Linz, Austria; 9 Valve Center, IRCCS Ospedale San Raaffaele, University Vita Salute, 20132 Milano, Italy; 10 Department of Cardiac Surgery and Heart Transplantation, Azienda Ospedaliera San Camillo Forlanini, 00152 Rome, Italy; 11 Division of Cardiac Surgery, Spedali Civili di Brescia, University of Brescia, 25121 Brescia, Italy; 12 Cardiothoracic and Vascular Department, University of Pisa, 56126 Pisa, Italy; 13 Servicio Cirugía Cardíaca, Hospital Álvaro Cunqueiro, 36312 Vigo, Spain; 14 Department of Cardiac Surgery, Inselspital University Hospital Bern, 3010 Bern, Switzerland; 15 Department of Cardiovascular and Thoracic Surgery, Cliniques Universitaires Saint Luc, 1200 Brussels, Belgium; 16 Department of Cardiovascular Medicine, University Hospital Leuven, 3000 Leuven, Belgium; 17 DZHK (German Center for Cardiovascular Research), Partner Site Munich Heart Alliance, 80336 Munich, Germany; 18 Department of Cardiac Surgery, Medical University of Graz, 8010 Graz, Austria

**Keywords:** mitral valve, MAC, mitral annular calcification, transcatheter mitral valve replacement, transcatheter treatments

## Abstract

**Objectives**: This study aims to review short- to intermediate-term outcomes after transapical transcatheter mitral valve replacement (TMVR) using the Tendyne valve system in patients with mitral annular calcification (MAC), including off-label use in severe MAC. **Methods**: This retrospective sub-analysis of the multicenter Tendyne European Experience (TENDER) registry included fifty-three MAC patients who underwent commercial Tendyne-TMVR in 15 European heart centers between 01/2020 and 06/2022. Patients were assigned to the mild (n = 16), moderate (n = 17), and severe MAC (n = 20) cohorts according to Guerrero’s MAC score. Additionally, the predictive value of detailed computed tomography-derived, quantitative, and qualitative MAC characteristics on clinical outcome was tested. **Results**: In this overall multimorbid patient population, predominantly treated for severe mitral regurgitation (MR), technical success rates were comparable among cohorts (mild MAC: 93.8% vs. moderate MAC: 88.2%vs. severe MAC: 95%, *p* = 0.720). Complete MR abolishment was achieved in 88.7% of patients, with no significant difference between cohorts in the incidence of residual MR >1+ (n = 1 in moderate MAC; *p* = 0.350) or paravalvular leakage >1+ (PVL; n = 2 in moderate MAC, *p* = 0.118) at discharge. All three in-hospital deaths occurred in patients with moderate MAC (*p* = 0.034). There were no significant differences in 1-year cardiovascular mortality (mild MAC: 23.1% vs. moderate MAC: 6.3% vs. severe MAC: 0%, *p* = 0.085) and overall mortality (mild MAC: 38.5% vs. moderate MAC: 43.8% vs. severe MAC: 18.8%, *p* = 0.291) between the cohorts, including in patients with off-label severe MAC. The rate of heart failure hospitalization at 1 year was significantly higher in the moderate MAC cohort (mild MAC: 10% vs. moderate MAC: 61.5%, severe MAC: 21.4%, *p* = 0.017). Further quantitative and qualitative MAC parameters showed no significant impact on 1-year survival or hemodynamic prosthetic performance. **Conclusions**: This MAC-focused analysis suggests that Valve-in-MAC using the Tendyne valve system is safe, technically feasible, and associated with satisfying hemodynamic and clinical outcomes, irrespective of MAC morphology.

## 1. Introduction

Calcific mitral valve (MV) disease is a common finding in patients with one or more cardiovascular risk factors (e.g., age, diabetes, and chronic kidney disease) [1], and is—particularly when causing elevated trans-mitral valve mean pressure gradients (MPG) [2]—associated with an increased risk of cardiovascular events and mortality [3].

Though surgical MV repair or replacement has been reported to be justifiable also in severe mitral annular calcification (MAC) [4], a substantial proportion of this patient population is multimorbid and therefore at prohibitive surgical risk. Therefore, less invasive transcatheter therapies are often the preferred strategy, and these patients are frequently the subject of complex heart team evaluations.

The role of transcatheter MV repair, such as edge-to-edge repair (TEER), in severe MAC management is restricted due to increased risk of unsatisfying hemodynamic results and subsequent low clinical–functional improvement [5]. Hence, transcatheter mitral valve replacement (TMVR) often represents the last resort in the presence of severe MAC.

Transapical (TA) Tendyne-TMVR is frequently applied since its CE/FDA approval but it is not certified for use in patients with severe MAC. Nevertheless, real-world data from the multicenter TENDER registry and results from the SUMMIT-MAC study demonstrate satisfying safety and efficacy outcomes, including symptomatic improvement [6,7,8,9]. The impact of MAC distribution and the MAC quantity on patient outcomes in the Tendyne-valve-in-MAC (ViMAC) is unclear and is the subject of investigation within this MAC-focused TENDER sub-analysis.

## 2. Materials and Methods

The present study represents a retrospective, exploratory MAC-specific sub-analysis of the TENDER observational study (TENDER, ClinicalTrials.gov Identifier: NCT04898335), which included patients from 15 European heart centers who underwent commercial TA-TMVR with the Tendyne valve system between January 2020 and June 2022.

### 2.1. Study Population and Cohort Definition

**Figure 1:**
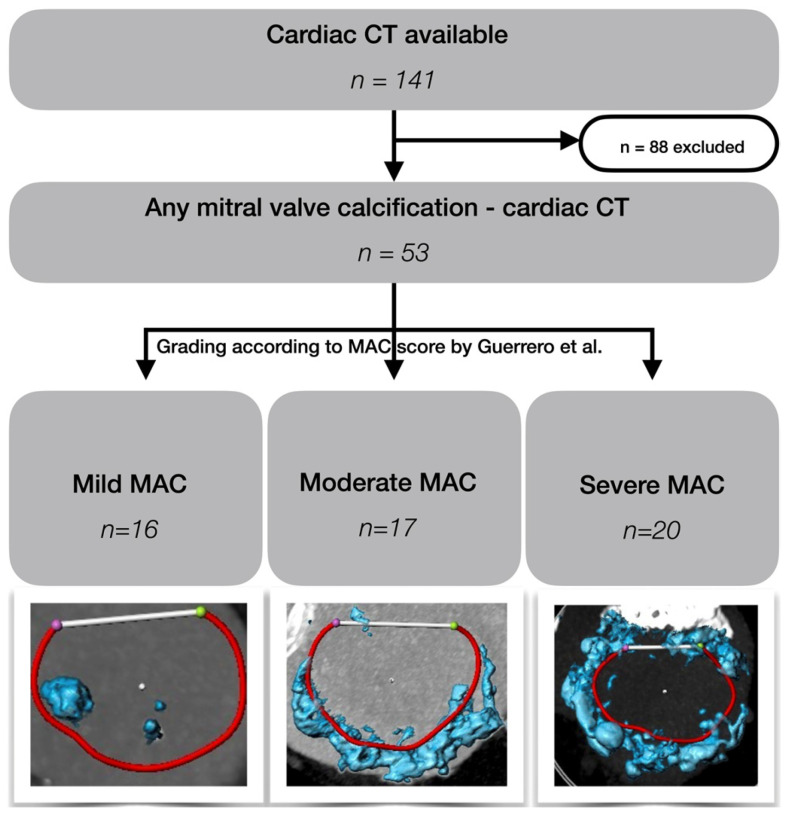
Flow chart of this MAC-specific TENDER sub-analysis. Cohort assignment according to cardiac computed tomography-derived MAC score by Guerrero et al. [10] in patients with mild, moderate, and severe MAC. CT—computed tomography, MAC—mitral annular calcification.

**Figure 2:**
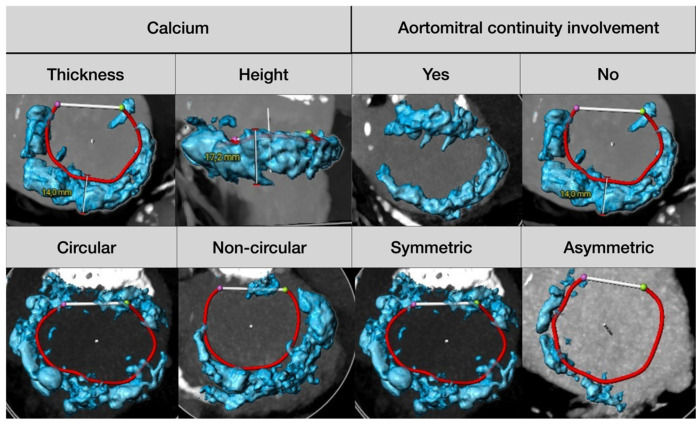
CT-derived MAC parameters. Insights in assessment of quantitative and qualitative MAC patterns. CT—computed tomography, MAC—mitral annular calcification.

The procedural planning and electrocardiogram-gated cardiac computed tomography (CT) scans of 141 patients were pseudonymized and provided to the Department of Cardiac Surgery, Medical University of Vienna, for retrospective review. Calculations were performed by one physician experienced in TMVR screening (>150 screening cases) using the mitral workflow of 3mensio (version 10.3, Pie Medical Imaging B.V., Maastricht, Netherlands). Patients without MV calcification were excluded, and patients with any calcific degeneration were assigned, applying the MAC score by Guerrero et al. [10], to either the mild (n = 16), moderate (n = 17), or severe MAC cohort (n = 20; Figure 1). Further MAC-specific calculations included: thickness, height, distribution, symmetry, and infestation into the aortomitral continuity (Figure 2).

### 2.2. Data Acquisition

Within the short- and mid-term results report of Wild and Hell et al., data acquisition was described in detail previously [6,7].

### 2.3. Valve-in-MAC Tendyne TMVR

**Figure A1:**
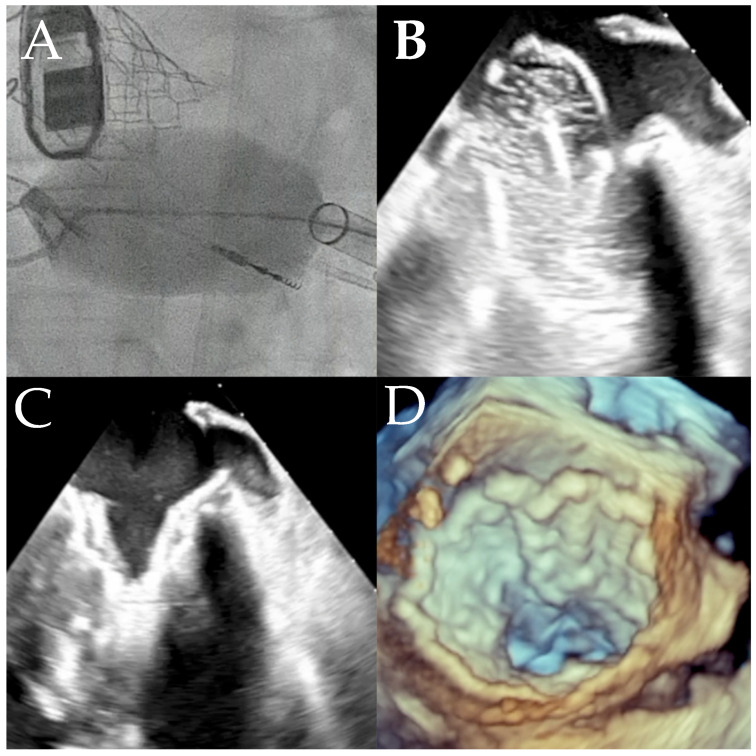
Balloon valvuloplasty in transapical transcatheter mitral valve replacement prior to deployment of the Tendyne prosthesis. (**A**) Fluoroscopy and transesophageal echocardiography (TOE); (**B**) during inflation of the non-compliant balloon. Following self-expansion of the prosthesis under TOE guidance (here: intercommissural view; (**C**) with visualization of the result by 3-dimensional “en face” view (**D**).

Procedural steps and considerations in treating severe MAC by TA-TMVR with the Tendyne valve system have been reported previously [11]. Contrary to standard label anatomy, balloon valvuloplasty (BAV)—depending on the MAC amount, residual MV area, and the surgeons’ preference—may be necessary to enable adequate self-expansion of the D-shaped bio-prosthesis. Therefore, an additional apical crossing with a large-bore introducer sheath for the introduction of the valvuloplasty balloon is necessary (Figure A1).

### 2.4. Endpoint Definition

Endpoint assessment was performed according to the current recommendations of the Mitral Valve Academic Research Consortium consensus statement [12].

### 2.5. Statistical Analysis

Continuous data were reported as the median and interquartile range, and categorical data as the absolute count and relative frequency.

Demographic, procedural, and outcome data were compared between the MAC cohorts. Therefore, the Kruskal–Wallis test for continuous variables and the *χ*^2^ for categorical data were applied.

Logistic regression models were built to estimate the impact of the MAC anatomy on device-related adverse outcomes (PVL > 1+, MV MPG ≥ 5 mmHg) and 1-year cardiovascular as well as 1-year overall mortality. Variables with *p* < 0.100 in the univariable model were further considered within the multivariable model.

Kaplan–Meier curves were generated to estimate 1-year all-cause and 1-year cardiovascular survival (95% confidence intervals [CI]), and differences between the cohorts were compared using the log-rank test.

For data analysis, Microsoft Excel 16.74, R 4.5.3, and SPSS 29.0 for MacIntosh were used.

Generally, a two-sided *p*-value ≤ 0.05 was considered statistically significant. Effect estimates are presented as odds ratios (OR) with 95% CI.

## 3. Results

### 3.1. Baseline Data

#### 3.1.1. Demographic Data

Demographic data from 53 patients, stratified according to MAC severity and summarized in Table 1, illustrate the multimorbidity of the overall study population, with no statistically significant differences observed between the cohorts. Overall, 73.6% of patients underwent prior cardiac surgery and/or intervention. This included one patient after surgical MV repair failure (annuloplasty) and four patients after TEER in the mitral position (n = 3: no device implanted, n = 1: single leaflet device attachment; all in the moderate or severe MAC cohorts).

#### 3.1.2. Echocardiographic Characteristics

MR was the leading indication for TA-TMVR (92.5%). Fifteen patients exhibited an elevated MV MPG > 5 mmHg, including two patients who underwent treatment for severe mitral stenosis. Baseline echocardiography assessed predominantly normally sized left ventricles with preserved/mildly reduced systolic function and severe pulmonary hypertension (Table 2). Tricuspid regurgitation ≥ 3+ and impaired right ventricular function were present in 26.4% and 32.1% of patients, respectively (for all parameters *p* > 0.05 between the cohorts).

#### 3.1.3. MAC-Specific CT-Calculations

MV calcification volume, height, and thickness ranged from 38 to 10,761.8 mm^3^, 2 to 27.8 mm and 2.9 to 20.9 mm, respectively (all *p* < 0.05 between the cohorts, Table 3). According to the considered parameters within the Guerrero MAC score, the incidence of leaflet calcification, circular and symmetric calcification, and bi-leaflet involvement differed significantly between the cohorts (*p* < 0.05).

### 3.2. TMVR Outcome

#### 3.2.1. Procedural Characteristics

TA-Tendyne-TMVR was associated with a high technical success rate irrespective of MAC severity: 92.5% in the overall study population and 95% in off-label, severe MAC. BAV to facilitate appropriate valve deployment was performed in 50% of patients with severe MAC, 35.2% of those with moderate MAC, and 6.3% of patients with mild calcifications (*p* = 0.019, Table 4). Immediate procedure-related adverse events, such as the incidence of major apical access complications, the need for valve retrieval, or the incidence of left ventricular outflow tract obstruction, were comparable between the cohorts. Procedural mortality was 0%.

#### 3.2.2. In-Hospital Course and Clinical 30-Day Follow-Up

The overall in-hospital and 30-day mortality rate was 5.7%. All three deaths occurred in patients treated for moderate MAC and were exclusively attributable to lethal sepsis (mild MAC: 0% vs. moderate MAC: 17.6% vs. severe MAC: 0%, *p* = 0.034; Table 5).

All three re-interventions at 30-day follow-up were indicated due to major apical access complications during the index hospitalization (mild MAC: 0% vs. moderate MAC: 11.8% vs. severe MAC: 5%, *p* = 0.442).

#### 3.2.3. Short-Term Echocardiographic Outcome

At discharge, complete MR elimination and freedom from PVL were recorded in overall 88.7% and 90.6% of the study population, respectively, and the incidence was comparable between the cohorts (Table 6).

An elevated MV MPG (≥5 mmHg) was observed in 35.9% of the treated patients. An increased MV MPG was numerically, but not statistically, more frequent in the moderate MAC cohort (mild MAC: 3.4 [2.6; 5] vs. moderate MAC: 5 [4; 6] vs. severe MAC: 4 [3.7; 5.2] mmHg; *p* = 0.078).

Logistic regression models revealed no statistically significant unfavorable effect of the quantitative and qualitative MAC parameters or MV size on hemodynamic prosthesis performance (MV MPG ≥ 5 mmHg) and the incidence of PVL > 1 (Table A1 and Table A2).

#### 3.2.4. One-Year Follow-Up and Survival Analysis

One-year survival data was available in 45 of 53 patients. Freedom from all-cause mortality and cardiovascular mortality at one year after TA-TMVR was 66.7% and 91.1%, respectively. All-cause and cardiovascular mortality showed no significant difference between patients with mild (38.5% and 23.1%), moderate (43.8% and 6.3%), and severe MAC (18.8% and 0%; both *p* > 0.05; Table 7).

In addition to three re-interventions related to access complications, two re-interventions were observed: (1) re-tethering on post-operative day 86 and (2) surgical MV replacement on post-operative day 272 due to prosthetic endocarditis.

Symptomatic improvement was less in the moderate MAC cohort: 45.5% (vs. 0% [mild MAC] vs. 18.2% [severe MAC], *p* = 0.075) and 61.5% (vs. 10% [mild MAC] vs. 21.4% [severe MAC], *p* = 0.017) of those patients were in NYHA > II and underwent heart failure hospitalization during the 1-year follow-up, respectively.

MAC amount and distribution, but also pre-BAV and baseline MV MPG, had no significant impact on all-cause and cardiovascular 1-year mortality (Table A3 and Table A4).

## 4. Discussion

We herein report the results of a MAC-specific sub-analysis of the European, multicenter, investigator-initiated TENDER registry. The results from our real-world experience can be summarized as follows:

(1) In an advanced sick patient collective with MAC and limited therapeutic options, TA-Tendyne-TMVR was—regardless of the MAC severity—technically feasible and safe (procedural-mortality: 0%).
(2) Increasing MAC severity necessitated higher rates of balloon valvuloplasty. This additional procedural maneuver seems not to be associated with higher rates of technical failure.
(3) Short-term hemodynamic prosthetic performance was comparable between patients off-label treated for severe MAC and those for on-label mild/moderate MAC anatomy.
(4) There was no statistically significant difference in 1-year overall and 1-year cardiovascular survival between MAC severity cohorts.
(5) The regression models suggest no impact of the annular dimension, MAC volume, its distribution, and symmetry on the incidence of post-procedural PVL >1+ or MV MPG ≥ 5 mmHg, or intermediate-term survival.
(6) The less symptomatic improvement observed in the moderate MAC cohort is most likely attributable to random variation in the context of a limited sample size, rather than indicating a distinct anatomical risk profile. Despite this, the finding is reported transparently and should be interpreted within the constraints of the study design.
(7) Predominantly low-profile valves (>75%) were implanted.

### 4.1. MAC—Treatment Principles

MAC is associated with multiple chronic, pro-atherosclerotic diseases and risk factors [1,2,3], necessitating careful consideration within the individualized heart team evaluations when deciding regarding a surgical, interventional, or conservative treatment strategy—a decision not supported by data from randomized controlled trials.

Reported short-term mortality and complication rates vary widely among patients undergoing MV surgery for severe MAC [13]. Compared with non-MAC patients, higher post-operative complication rates and short-term mortality have been reported [14,15]. However, clinical outcomes appear to depend also on patient selection and surgical experience. For example, Uchimuro et al. reported 0% in-hospital mortality and, after accounting for the patient multimorbidity, an acceptable 5-year survival of 75.6% in a cohort with a mean age of 70 years undergoing surgery for severe MAC [16]. In general, the positive impact of center volume—as a surrogate of surgical experience—on the outcome after MV surgery has been recognized [14,17], and may be even more pronounced in severe MAC, because specific expertise in managing the calcification, decision-making between “resect or respect” and replacement-versus-repair strategy, respectively, and, when necessary, advanced skills in mitral annular reconstruction are required [13,18].

Transcatheter MV edge-to-edge repair plays a minor role in MAC management, mainly due to the risk of iatrogenic mitral valve stenosis, subsequently lacking symptomatic improvement and high 1-year mortality (>40%) [5].

The MAC-specific treatment algorithm proposed by Chehab et al. recommends first-line screening for off-label TMVR [19], which is discussed in detail herein.

### 4.2. Comparison Tendyne ViMAC and Sapien ViMAC (Excluding M3)

Severe MAC represents an off-label indication for dedicated TMVR devices, such as the Tendyne valve system, due to the global feasibility trial inclusion and exclusion criteria [20]. However, previous reports—including the recently published prospective SUMMIT-MAC clinical trial—homogenously report high technical safety, nearly complete MR elimination, and a low PVL rate along with symptomatic improvement and a reduction in heart failure hospitalization, especially in this patient population [6,7,8,9,21,22]. Our analysis adds that MAC morphology and severity do not impact clinical and hemodynamic outcomes. In summary, these encouraging experiences suggest a shift in clinical practice away from automatically excluding patients with severe, symptomatic mitral valve disease despite optimized medical therapy based solely on MAC severity, toward a more liberal off-label use of Tendyne ViMAC TMVR. This approach aligns with an individualized, patient-specific therapeutic strategy.

The Sapien valve system is the longest investigated device system (Sapien XT—S3) for use in severe MAC anatomies [10,23,24]. Table 8 offers a comparison of baseline and procedural characteristics as well as short- to intermediate-term outcomes between the largest experience with Sapien ViMAC TMVR (MAC Global Registry) and our experience in severe MAC (cohort C) [24]. These comparably sick patient collectives underwent ViMAC TMVR for different leading MV pathologies (MAC Global Registry: 96% mitral stenosis; TENDER-MAC: 90% MR, 55% stenotic MAC). The MAC Global Registry did not report details on MAC morphology, but Guerrero et al. described in a Sapien ViMAC experience less calcium thickness (8.3 vs. 14.2 mm) and height (11.6 vs. 16.5 mm) compared to our analysis [10].

Our MAC-TENDER analysis reports favorable technical, hemodynamic, safety, efficacy, and survival outcome: in detail, Sapien ViMAC was related to a higher rate of procedure-related adverse events (left ventricular outflow tract [LVOT] obstruction: 11% vs. 0%; valve embolization: 4% vs. 0%; need for a second valve: 15% vs. 0%), a higher rate of PVL (any PVL ≥ 1: 33% vs. 15%), and the incidence of hemolysis (4% vs. 0%) as well as a higher MV re-intervention rate at 1 year, likely contributing to the relevant difference in 1-year cardiovascular (24% vs. 0%) and overall mortality (54% vs. 0%).

The adverse event rate following Sapien ViMAC was independent of the access route (trans-septal, TA, or hybrid trans-atrial) and is rather resulting from technical issues related to the balloon-expansion valve design, limited predictability of valve anchoring, and of the final implantation height [25], all of which are not present when using the self-expandable, tether-pad-fixated Tendyne prosthesis [6,7,8,9,26]. Further, the Sapien valve system (excluding M3) is literally designed for use in the aortic position; the round prosthesis does, therefore—contrarily to the dedicated D-shaped TMVR device—not seal the commissures and is thus prone to PVL [13,23,24].

### 4.3. Tendyne ViMAC TMVR—Technical Considerations and Limitations

Especially in the first Tendyne ViMAC cases, the evaluation of the necessity for pre-BAV, which means an additional maneuver with a large-bore sheath through the LV apex [11], can be challenging due to a lack of recommendations. This decision is mainly driven by CT-derived MV assessment. A small MV area or a protruding calcium spur have been discussed as driving factors for pre-ballooning [8]. We found a strong relation between BAV performance and the MAC score by Guerrero (MAC-score mild/moderate/severe: OR 3.11, CI 1.33–7.29, *p* = 0.009) and the quantitative MV calcification volume (OR 1.00, CI 1.00–1.00; *p* = 0.008), respectively; both are simple parameters to rely on—particularly during the center’s initial program phase.

BAV was predominantly performed using a non-compliant balloon, sized according to the CT-derived anterior-to-posterior diameter to minimize the risk of injury to the MV annulus and the aortomitral continuity, respectively. With this approach, no unintended tissue injury (e.g., annular rupture) was observed, and the intermediate-term cardiovascular and overall mortality were not increased after BAV (cardiovascular 1-year mortality: 5.9% [BAV] vs. 8.3% [no BAV]; post hoc χ^2^: *p* = 0.753; all-cause 1-year mortality: 17.7% [BAV] vs. 33.3% [no BAV]; post hoc χ^2^: *p* = 0.237).

TMVR screening for anatomical suitability is performed using multimodal imaging, including echocardiography and CT. As our data come from the initial program phase of Tendyne-TMVR in Europe, there may have been a particular focus on selecting patients with anatomically favorable profiles, especially among those accepted for compassionate use in severe MAC anatomies.

Our previous surgical TENDER sub-analysis identified apical access complications and sepsis as the leading reasons for short-term mortality after TA-Tendyne-TMVR [26]. These adverse events might be partially preventable by further improving clinical and anatomical patient selection (e.g., apical myocardial thickness), reducing the incidence of LVOT obstruction (e.g., the MitraCut technique [27,28,29]), and surgical training.

Contrary to our experience, Coisne et al.—including patients undergoing TA and trans-septal TMVR with different dedicated TMVR devices—reported a significantly higher incidence of access complications, bleeding events, and post-interventional kidney injury stage 2/3 in patients with moderate/severe MAC than in those with none/mild MAC. Nevertheless, 2-year all-cause and 2-year cardiovascular mortality were comparable between both cohorts [22].

Less than one-third of patients are anatomically eligible for TMVR, mainly due to unfitting annular dimensions, a small left ventricle, and/or a given risk of LVOT obstruction [30]. In this context, Ludwig et al. identified women with degenerative MR, MAC, and preserved systolic LV function—next to multimorbid patients with secondary MR—as a critical cluster with almost no therapeutic scope and a need for technical innovations [31].

In this context, addressing the anterior mitral leaflet, e.g., by the MitraCut technique, might enable TMVR also in patients with borderline-sized LVOT [27,28,29].

There are two Tendyne valve profiles: standard (larger orifice area approximately: 3.0 cm^2^) and low (reduced height, orifice area approximately: 2.2 cm^2^, to minimize LVOT obstruction). Selection of the low-profile valve—as in >75% of our study population—is necessary in most patients with degenerative MR and normal-to-small LV dimensions. This likely explains the relatively high incidence of MV MPG ≥5 mmHg at discharge observed in our analysis. Larger cohort studies are warranted to investigate the impact of periprosthetic MV MPG on patient survival and functional outcomes.

### 4.4. Future Outlook in ViMAC TMVR

In 2025, a dedicated, trans-septal Sapien TMVR device (Sapien M3, Edwards LifeScience, Irvine, CA, USA) was introduced and CE/FDA approved. Technical refinements, including a novel sub-valvular anchoring system and the dock, may allow for use in non-calcific MV disease but may also improve outcomes in the ViMAC setting. However, clinical data with this device are currently limited to results from the main cohort of the single-arm, prospective ENCIRCLE trial, which excluded patients with severe MAC [32]. Results from the MAC cohort are awaited soon.

### 4.5. Limitations

This study is subject to limitations. First, it used an exploratory study design. Second, the sample size is limited, particularly due to the off-label nature of this therapeutic approach, although it represents, alongside the CHOICE-MI MAC analysis and the SUMMIT-MAC trial, the largest experience in this specific patient population to date. Third, follow-up was occasionally incomplete, partly due to the COVID-19 pandemic and reduced outpatient contact from local access restrictions.

## 5. Conclusions

TA-Tendyne TMVR is safe, provides reliable MR reduction, and results in satisfying clinical outcomes regardless of the MAC amount or morphology. Accordingly, ViMAC Tendyne TMVR may offer a valuable therapeutic option for carefully selected high-risk MAC patients.

## Figures and Tables

**Table 1:** Demographic data.

	Mild Mitral Annular Calcification (n = 16)	Moderate Mitral Annular Calcification (n = 17)	Severe Mitral Annular Calcification (n = 20)	*p*-Value
Female, n (%)	10 (62.5)	6 (35.3)	7 (35)	0.182
Age, years	76 [72; 82]	77 [72; 82]	79 [70; 83]	0.998
STS Prom, %	5.1 [3.3; 7.4]	7.3 [3.8; 9.7]	5.1 [3.2; 9.3]	0.440
EuroScore II, %	6.1 [2.8; 11.5]	7.4 [3.9; 8.2]	4.7 [2.3; 11.1]	0.687
NYHA stadium, III-IV	16 (100)	14 (82.2)	20 (100)	0.165
Previous HFH, n (%)	9 (56.3)	10 (58.8)	11 (55)	0.951
NT-proBNP, ng·mL^−1^	2852 [1496; 7863]	3630 [1708; 6854]	7443 [2892; 12,097]	0.086
BMI, kg·m^−2^	27.6 [24.1; 32.1]	26.2 [24; 29.1]	26.1 [22.8; 28.7]	0.709
eGFR, mL·min^−1^	42 [30; 57]	41 [26; 67]	37 [25; 58]	0.863
COPD, n (%)	4 (25)	5 (29.4)	2 (10)	0.308
Atrial fibrillation/fluttern, n (%)	8 (50)	15 (88.2)	13 (65)	0.059
Coronary artery disease, n (%)	10 (62.5)	13 (76.5)	13 (65)	0.649
Previous PCI, n (%)	6 (37.5)	7 (41.2)	6 (30)	0.769
CABG, n (%)	5 (31.3)	7 (41.2)	6 (30)	0.746
SAVR or TAVR, n (%)	4 (25)	8 (47)	16 (80)	0.220
SAVR, n (%)	2 (12.5)	5 (29.4)	8 (40)	
TAVR, n (%)	2 (12.5)	3 (17.6)	8 (40)	
Previous MV surgery/intervention, n (%)	3 (18.8)	2 (11.8)	0 (0)	0.277
Surgical repair, n (%)	1 (6.3)	0 (0)	0 (0)	
TEER, n (%)	2 (12.5)	2 (11.8)	0 (0)	
Pacemaker/ICD, n (%)	2 (12.5)	3 (17.6)	4 (20)	0.834
Heart failure medication/device				
ACE-inhibitor/ARB, n (%)	6 (37.5)	9 (52.9)	15 (75)	0.104
Beta-blocker, n (%)	13 (81.3)	16 (94.1)	15 (75)	0.266
Diuretics, n (%)	12 (75)	16 (94.1)	15 (75)	0.293
CRT, n (%)	2 (12.5)	0 (0)	1 (5)	0.296

ACE—angiotensin converting enzyme; ARB—angiotensin receptor blocker; BMI—body mass index; CABG—coronary artery bypass grafting; COPD—chronic obstructive pulmonary disease; CRT—cardiac resynchronization device; eGFR—estimated glomerular filtration rate; HFH—heart failure hospitalization; ICD—implantable cardioverter defibrillator; MV—mitral valve; NT-proBNP—N-terminal pro brain natriuretic peptide; NYHA—New York Heart Association class; PCI—percutaneous coronary intervention; SAVR—surgical aortic valve replacement; TAVR—transcatheter aortic valve replacement; TEER—transcatheter edge-to-edge repair.

**Table 2:** Echocardiographic characteristics.

	Mild Mitral Annular Calcification (n = 16)	Moderate Mitral Annular Calcification (n = 17)	Severe Mitral Annular Calcification (n = 20)	*p*-Value
LV ejection fraction, %	54.5 [47; 57.8]	50 [45; 60.5]	56 [45.5; 60]	0.755
LVEDD, mm	52.5 [46.3; 59.3]	52 [49; 57]	48 [46; 61.5]	0.891
MR 3+/4+	16 (100)	15 (88.2)	18 (90)	0.431
MV MPG, mmHg	4 [3; 5]	4 [3; 5]	5 [2; 7.1]	0.883
MV MPG 5–10 mmHg	3 (18.8)	2 (13.3)	8 (42.1)	
MV MPG > 10 mmHg	0 (0)	1 (6.7)	1 (5.3)	
MV disease—mechanism				0.155
Primary MV disease, n (%)	8 (50)	8 (47.1)	14 (70)	
Secondary MV disease, n (%)	4 (25)	2 (11.8)	0 (0)	
Mixed MV disease, n (%)	4 (25)	7 (41.2)	6 (30)	
TAPSE < 17 mm, n (%)	5 (31.3)	6 (37.5)	6 (30)	0.883
TR ≥ III	1 (6.7)	5 (29.4)	8 (40)	0.105
estimated sPAP, mmHg	50.1 [42.3; 60.3]	50 [40; 62]	60 [47; 66.8]	0.390

LV—left ventricular; LVEDD—left ventricular end-diastolic diameter; MPG—mean pressure gradient; MR—mitral regurgitation; MV—mitral valve; sPAP—systolic pulmonary artery pressure; TAPSE—tricuspid annular plane systolic excursion.

**Table 3:** CT-derived mitral valve characteristics.

	Mild Mitral Annular Calcification (n = 16)	Moderate Mitral Annular Calcification (n = 17)	Severe Mitral Annular Calcification (n = 20)	*p*-Value
MV annulus, A/P, mm	30.1 [27.9; 34]	28.7 [26.7; 31.9]	29 [24.5; 31.7]	0.482
MV annulus, IC, mm	39.7 [35.7; 42.8]	39.9 [36; 41.2]	38.9 [37; 42]	0.762
MV perimeter, mm^2^	118.2 [108.5; 127.6]	115.4 [106.3; 122.8]	115.8 [107.5; 121.5]	0.732
MAC volume, mm^3^	247.5 [89.3; 462.3]	476.8 [256.1; 1419.5]	4770.6 [2010.5; 6630.5]	<0.001 *
Maximal calcium thickness, mm	5 [3.4; 9]	10.5 [9.4; 15.5]	14.6 [10.9; 16.6]	<0.001 *
Maximal calcium height, mm	8.4 [4.8; 10]	8.5 [6.6; 15.9]	16.2 [13.9; 18.3]	<0.001 *
Leaflet calcification, n (%)	4 (25)	7 (41.2)	14 (70)	0.023 *
Calcified aortomitral continuity, n (%)	2 (12.5)	6 (35.3)	12 (60)	0.014 *
Calcium distribution				
Circular, n (%)	1 (6.3)	1 (5.9)	7 (35)	0.003 *
Spotty, n (%)	15 (93.8)	15 (88.2)	8 (40)	
Circular and spotty, n (%)	0 (0)	1 (5.9)	5 (25)	
Symmetric, n (%)	1 (6.3)	1 (5.9)	8 (40)	0.009 *
Asymmetric, n (%)	15 (93.8)	16 (94.1)	12 (60)	
Predominantly anterior, n (%)	3 (18.8)	6 (35.3)	4 (20)	0.125
Predominantly posterior, n (%)	12 (75)	9 (52.9)	9 (45)	

* *p* < 0.05. A/P—anterior to posterior diameter; IC—intercommissural diameter; MAC—mitral annular calcification; MV—mitral valve; sPAP—systolic pulmonary artery pressure; TAPSE—tricuspid annular plane systolic excursion.

**Table 4:** Procedural characteristics.

	Mild Mitral Annular Calcification (n = 16)	Moderate Mitral Annular Calcification (n = 17)	Severe Mitral Annular Calcification (n = 20)	*p*-Value
Procedural time, min	122 [105; 145]	126 [113; 157]	125 [96; 169]	0.948
Technical success, n (%)	15 (93.8)	15 (88.2)	19 (95)	0.720
Balloon valvuloplasty, n (%)	1 (6.3)	6 (35.2)	10 (50)	0.019 *
Low-profile valve, n (%)	12 (75)	14 (82.4)	14 (70)	0.679
Valve implanted, n (%)	15 (93.8)	17 (100)	20 (100)	0.308
Valve retrieval, n (%)	2 (12.5)	1 (5.9)	2 (10)	0.805
LVOT obstruction, n (%)	2 (12.5)	3 (17.6)	0 (0)	0.165
Major access complication, n (%)	0 (0)	2 (11.8)	1 (5)	0.339
ECMO/heart-lung machine, n (%)	0 (0)	0 (0)	1 (5)	0.431
Intra-procedural mortality, n (%)	0 (0)	0 (0)	0 (0)	1.000
Procedural mortality, n (%)	0 (0)	0 (0)	0 (0)	1.000

* *p* < 0.05. ECMO—extracorporeal membrane oxygenation; LVOT—left ventricular outflow tract.

**Table 5:** In-hospital and 30-day follow-up.

	Mild Mitral Annular Calcification (n = 16)	Moderate Mitral Annular Calcification (n = 17)	Severe Mitral Annular Calcification (n = 20)	*p*-Value
*In-hospital follow-up*				
Red blood cell units, n	0 [0; 3]	0.5 [0; 2]	0 [0; 2]	0.860
Life-threatening bleeding, n (%)	1 (6.3)	2 (11.8)	1 (5)	0.356
Bleeding BARC 2/3/5, n (%)	3 (18.3)	6 (35.2)	3 (15)	0.307
Sepsis, n (%)	2 (12.5)	3 (17.6)	0 (0)	0.163
Lethal sepsis, n (%)	0 (0)	3 (17.6)	0 (0)	0.034 *
Acute kidney injury, n (%)	3 (18.3)	6 (35.2)	3 (15.8)	0.360
Dialysis, n (%)	1 (6.3)	1 (5.9)	0 (0)	0.536
Intensive care unit, days	3 [1; 4]	2 [1; 15]	1.5 [1; 3]	0.419
Length of index hospitalization, days	11 [10; 14]	14 [9; 20]	13 [9; 16]	0.771
In-hospital mortality, n (%)	0 (0)	3 (17.6)	0 (0)	0.034 *
				
*30-day follow-up*	*(15/16)*	*(17/17)*	*(19/20)*	
All-cause mortality	0 (0)	3 (17.6)	0 (0)	0.038 *
Re-Intervention rate, n (%)	0 (0)	2 (11.8)	1 (5)	0.356
Stroke, n (%)	0 (0)	0 (0)	1 (5)	0.442
Myocardial infarction, n (%)	0 (0)	0 (0)	1 (5)	0.442

* *p* < 0.05. BARC—bleeding academic research consortium.

**Table 6:** Echocardiographic results (discharge).

	Mild Mitral Annular Calcification (n = 16)	Moderate Mitral Annular Calcification (n = 17)	Severe Mitral Annular Calcification (n = 20)	*p*-Value
Paravalvular leakage				
I, n (%)	1 (6.3)	1 (5.9)	1 (5)	0.978
≥2, n (%)	0 (0)	2 (11.8)	0 (0)	0.118
Mitral regurgitation				
I, n (%)	0 (0)	2 (11.8)	3 (15)	0.308
≥2, n (%)	0 (0)	1 (5.9)	0 (0)	0.350
Mitral valve mean pressure gradient				
mmHg	3.4 [2.6; 5]	5 [4; 6]	4 [3.7; 5.2]	0.078
≥5 mmHg, n (%)	4 (25)	8 (47.1)	7 (35)	0.283

**Table 7:** One-year follow-up.

	Mild Mitral Annular Calcification (n = 16)	Moderate Mitral Annular Calcification (n = 17)	Severe Mitral Annular Calcification (n = 20)	*p*-Value
All-cause mortality, n (%)	5/13 (38.5)	7/16 (43.8)	3/16 (18.8)	0.291
Cardiovascular mortality, n (%)	3/13 (23.1)	1/16 (6.3)	0/16 (0)	0.085
Overall re-intervention rate, n (%)	1/12 (8.3)	3/14 (21.4)	1/15 (6.7)	0.425
Re-intervention rate after discharge, n (%)	1/12 (8.3)	1/14 (7.1)	0/15 (0)	0.540
Stroke, n (%)	2/9 (22.2)	0/11 (0)	1/11 (9.1)	0.137
Disabling stroke, n (%)	2/9 (22.2)	0/11 (0)	0/11 (0)	
Myocardial infarction, n (%)	0/9 (0)	0/11 (0)	1/11 (9.1)	0.355
Hemolysis, n (%)	0/7 (0)	2/10 (20)	0/10 (0)	0.159
Valve thrombosis, n (%)	0/9 (0)	1/10 (10)	0/10 (0)	0.374
Valve migration/embolization, n (%)	0/9 (0)	1/10 (10)	0/10 (0)	0.374
NYHA, I–IV	2 [1; 2]	2 [2; 3]	2 [1; 2]	0.354
NYHA III/IV, n (%)	0/7 (0)	5/11 (45.5)	2/11 (18.2)	0.075
Heart failure hospitalization, n (%)	1/10 (10)	8/13 (61.5)	3/14 (21.4)	0.017 *

* *p* < 0.05. NYHA—New York Heart Association class.

**Table 8:** Sapien vs. Tendyne ViMAC TMVR.

	MAC Global Registry (n = 116)	TENDER Sub-Analysis— Severe MAC Cohort (n = 20)
***Baseline***		
Age, years	73 ± 12	76 ± 9
Female, %	68.1	35
Previous SAVR/TAVR, %	52.7	80
Previous CABG, %	32.1	30
LV-EF	60 ± 10	54 ± 9
MV MPG	11.5 ± 4.2	5.0 ± 3.0
***Procedural***		
Transapical TMVR, %	39.7	100
Concomitant SAVR/TAVR, %	15.2	0
Technical success, %	76,7	95
Relevant LVOT-obstruction, %	11.2	0
Application of LVOT-obstruction preventive technique, %	6	0
Valve embolization, %	4.3	0
Valve retrieval, %	0	10
Need for second valve, %	14.7	0
Conversion to bail-out open-heart surgery, %	3.5	5
Transprosthetic MV MPG, mmHg	4.4 ± 2.4	4.4 ± 1.4
Residual MR/PVL		
Trace or none, %	62.2	85
Mild, %	32.9	15
Moderate, %	0	0
≥3+, %	4.9	0
***30-day outcome***		
All-cause mortality, %	25	0
Cardiovascular mortality, %	12.9	0
Stroke, %	4.3	5.3
Myocardial infarction, %	0.9	5.3
MV re-intervention, %	7.8	0
Valve embolization, %	4.3	0
Valve migration, %	1.7	0
Endocarditis, %	0.9	0
Haemolytic anemia, %	3.5	0
Valve thrombosis, %	0	0
***1-year outcome***		
All-cause mortality, %	53.8	18.8
Cardiovascular mortality, %	23.6	0
Stroke, %	6.6	9.1
Myocardial infarction, %	1.9	9.1
MV re-intervention, %	12.3	0
Valve embolization, %	4.7	0
Valve migration, %	2.8	0
Endocarditis, %	3.8	0
Haemolytic anemia, %	3.8	0
Valve thrombosis, %	1.9	0

CABG—coronary artery bypass, grafting; LVOT—left ventricular outflow tract; MPG—mean pressure gradient; MR—mitral regurgitation; MV—mitral valve; PVL—paravalvular leakage; SAVR—surgical aortic valve replacement; TAVR—transcatheter aortic valve replacement; TMVR—transcatheter mitral valve replacement.

## Data Availability

The data presented in this study are available on request from the corresponding author.

## Abbreviations

The following abbreviations are used in this manuscript:

BAV	Balloon valvuloplasty
CT	Computed tomography
MAC	Mitral annular calcification
MPG	Mean pressure gradient
MV	Mitral valve
MR	Mitral regurgitation
LVOT	Left ventricular outflow tract
TA	Trans-apical
TEER	Transcatheter edge-to-edge repair
TENDER	TENDyne European experience Registry
TMVR	Transcatheter mitral valve replacement
ViMAC	Valve in mitral annular calcification

## Appendix A

**Table A1:** Univariable regression model—impact of MAC morphology on PVL > 1 (discharge).

	Odds Ratio	Confidence Interval	*p*-Value
MV annulus, A/P, mm	1.209	0.834–1.753	0.316
MV annulus, IC, mm	1.137	0.796–1.624	0.482
MV perimeter, mm^2^	1.064	0.933–1.215	0.354
MAC Guerrero, mild/moderate/severe	0.861	0.153–4.839	0.865
MAC quantification, mm^3^	1.000	1.000–1.001	0.430
Maximal calcium thickness, mm	1.290	0.877–1.897	0.196
Maximal calcium height, mm	1.056	0.848–1.314	0.627
Calcified aortomitral continuity, n (%)	1.632	0.096–27.648	0.735
Location of dominant calcification, anterior/posterior	0.814	0.119–5.556	0.833
Balloon valvuloplasty	2.125	0.125–36.182	0.602
MV MPG baseline	1.124	0.688–1.837	0.640

A/P—anterior to posterior diameter; IC—intercommissural diameter; MAC—mitral annular calcification; MPG—mean pressure gradient; MV—mitral valve.

**Table A2:** Univariable regression model—impact of MAC morphology on MV MPG ≥ 5 mmHg (discharge).

	Odds Ratio	Confidence Interval	*p*-Value
MV annulus, A/P, mm	0.986	0.846–1.150	0.856
MV annulus, IC, mm	1.055	0.923–1.206	0.430
MV perimeter, mm^2^	1.014	0.961–1.070	0.616
MAC Guerrero, mild/moderate/severe	1.107	0.871–1.407	0.407
MAC quantification, mm^3^	1.000	1.000–1.000	0.776
Maximal calcium thickness, mm	1.057	0.936–1.193	0.372
Maximal calcium height, mm	1.018	0.918–1.129	0.734
Calcified aortomitral continuity, n (%)	2.125	0.631–7.157	0.224
Location of dominant calcification, anterior/posterior	1.851	0.839–4.083	0.127
Balloon valvuloplasty	2.111	0.586–7.606	0.253
MV MPG baseline	1.118	0.888–1.407	0.342

A/P—anterior to posterior diameter; IC—intercommissural diameter; MAC—mitral annular calcification; MPG—mean pressure gradient; MV—mitral valve.

**Table A3:** Univariable regression model—impact of MAC morphology on 1-year cardiovascular mortality.

	Odds Ratio	Confidence Interval	*p*-Value
MV annulus, A/P, mm	0.971	0.746–1.263	0.825
MV annulus, IC, mm	0.992	0.794–1.239	0.945
MV perimeter, mm^2^	0.986	0.899–1.082	0.768
MAC Guerrero, mild/moderate/severe	0.156	0.020–1.210	0.076
MAC quantification, mm^3^	0.999	0.997–1.001	0.302
Maximal calcium thickness, mm	0.807	0.618–1.055	0.117
Maximal calcium height, mm	0.924	0.766–1.116	0.412
Calcified aortomitral continuity, n (%)	1.562	0.200–12.234	0.671
Location of dominant calcification, anterior/posterior	0.421	0.064–2.780	0.369
Balloon valvuloplasty	0.718	0.068–7.580	0.783
MV MPG baseline	1.090	0.741–1.602	0.662

A/P—anterior to posterior diameter; IC—intercommissural diameter; MAC—mitral annular calcification; MPG—mean pressure gradient; MV—mitral valve.

**Table A4:** Univariable regression model—impact of MAC morphology on 1-year overall mortality.

	Odds Ratio	Confidence Interval	*p*-Value
MV annulus, A/P, mm	0.997	0.851–1.169	0.972
MV annulus, IC, mm	1.085	0.931–1.264	0.297
MV perimeter, mm^2^	1.021	0.964–1.081	0.479
MAC Guerrero, mild/moderate/severe	0.622	0.282–1.374	0.240
MAC quantification, mm^3^	1.000	1.000–1.000	0.142
Maximal calcium thickness, mm	0.947	0.828–1.083	0.427
Maximal calcium height, mm	0.926	0.831–1.032	0.164
Calcified aortomitral continuity, n (%)	2.286	0.644–8.114	0.201
Location of dominant calcification, anterior/posterior	1.389	0.630–3.059	0.415
Balloon valvuloplasty	0.432	0.100–1.872	0.262
MV MPG baseline	0.954	0.733–1.241	0.724

A/P—anterior to posterior diameter; IC—intercommissural diameter; MAC—mitral annular calcification; MPG—mean pressure gradient; MV—mitral valve.
